# Mapping Topographic Structure in White Matter Pathways with Level Set Trees

**DOI:** 10.1371/journal.pone.0093344

**Published:** 2014-04-08

**Authors:** Brian P. Kent, Alessandro Rinaldo, Fang-Cheng Yeh, Timothy Verstynen

**Affiliations:** 1 Department of Statistics, Carnegie Mellon University, Pittsburgh, Pennsylvania, United States of America; 2 Department of Biomedical Engineering, Carnegie Mellon University, Pittsburgh, Pennsylvania, United States of America; 3 Department of Psychology and Center for the Neural Basis of Computation, Carnegie Mellon University, Pittsburgh, Pennsylvania, United States of America; University of Manchester, United Kingdom

## Abstract

Fiber tractography on diffusion imaging data offers rich potential for describing white matter pathways in the human brain, but characterizing the spatial organization in these large and complex data sets remains a challenge. We show that level set trees–which provide a concise representation of the hierarchical mode structure of probability density functions–offer a statistically-principled framework for visualizing and analyzing topography in fiber streamlines. Using diffusion spectrum imaging data collected on neurologically healthy controls (N = 30), we mapped white matter pathways from the cortex into the striatum using a deterministic tractography algorithm that estimates fiber bundles as dimensionless streamlines. Level set trees were used for interactive exploration of patterns in the endpoint distributions of the mapped fiber pathways and an efficient segmentation of the pathways that had empirical accuracy comparable to standard nonparametric clustering techniques. We show that level set trees can also be generalized to model pseudo-density functions in order to analyze a broader array of data types, including entire fiber streamlines. Finally, resampling methods show the reliability of the level set tree as a descriptive measure of topographic structure, illustrating its potential as a statistical descriptor in brain imaging analysis. These results highlight the broad applicability of level set trees for visualizing and analyzing high-dimensional data like fiber tractography output.

## Introduction

Fiber tractography on diffusion weighted imaging (DWI) data can provide a high-resolution map of the anatomical connections between two brain areas [1]. The deterministic variant of fiber tractography generates a set of simulated fiber streamlines that provide rich information about the topographic structure of white matter pathways [2]–[4]. This method has been used recently to characterize the sheet-like layout of large, myelinated pathways [5], map the organization of fiber bundles within the same pathway [6]–[8], identify novel neuroanatomical patterns [9]–[12] and quantify the global structural connectivity between large sets of brain regions [3], [13], providing a so-called structural “connectome” of the human brain (see Van Essen et al. (2012) [14]). The topography and connectivity of the structural connections identified with fiber tractography have also been shown to relate directly to corresponding functional connectivity [15] and task-evoked functional dynamics [6], [16], highlighting the relationship between structure and function in neural systems. Despite these advances, the lack of descriptive metrics for the spatial topography of white matter pathways remains a standing problem with structural connectivity analysis (see Jbabdi et al. (2013) [17]).

Clustering is a popular method for summarizing the spatial organization of white matter pathways [18], [19], but clustering is often a difficult and ill-defined task. Many of the proposed approaches, such as fuzzy c-means [20], [21], spectral clustering [22], [23], diffusion maps [24], local linear embedding [25], geometric clustering [26], [27] and white matter atlas matching [28], [29], assume there is a single well-defined partition of the data into *K* separate groups, where *K* is presumed known *a priori*. However, when the data are noisy or have a high degree of complexity or spatial heterogeneity, as is often the case in neuroimaging, it is more appropriate to assume the data have multi-scale clustering features that can be captured by a hierarchy of nested partitions of different sizes. These partitions and their hierarchy provide a wealth of information about the data beyond typical clustering results, unburdening the practitioner from the need to guess the “right” number of clusters, providing a global summary of the entire data set and offering the ability to select sub-clusters at different levels of spatial resolution depending on the scientific problem at hand.

There are many well-established hierarchical clustering methods, some of which have been applied to the problem of fiber track segmentation [30]–[33]. However, these methods often suffer from a lack of statistical justification. Single linkage clustering, for example, is known to be inconsistent in dimensions greater than one [34] and suffers from the problem of “chaining” [18]. In addition, the dendrograms that result from agglomerative hierarchical clustering do not indicate the optimal number of clusters; the practitioner must specify the desired number of clusters or a threshold at which to cut the dendrogram. Furthermore, the dendrograms that result from these methods are rarely used as statistical descriptors in their own right.

Several recent fiber clustering analyses propose more sophisticated methods that do not require *a priori* knowledge of the number of clusters. Wasserman and Deriche (2008) [35] and Zvitia et al. (2008) [36] use the mean-shift clustering algorithm, which finds clusters that correspond to the modes of an assumed probability density function. Brun et al. (2004) use spectral clustering but avoid choosing a cluster number by doing recursive binary data partitions [37]. Wang et al. (2011) use a hierarchical Bayesian mixture model over supervoxels to estimate white matter segmentation, with the number of clusters chosen automatically by a Dirichlet process [38]. Different clustering scales are achieved by defining supervoxels of various sizes. Many of these methods are capable of clustering at multiple data resolutions, but this is generally not the focus and the multi-scale clustering results are typically not exploited for further analysis.

In this article we introduce and apply the principles of high-density clustering [39] for complex fiber tractography from a high-angular resolution form of DWI. We implement a general procedure called the *level set tree* for accurate estimation of nested subsets of high-density data points. Like other agglomerative clustering methods, the output of our procedure is a hierarchy of clusters that can be represented with a dendrogram. But unlike any other hierarchical clustering method, the dendrogram obtained by the level set tree procedure has a direct probabilistic interpretation in terms of underlying probability density function (see next section for details and background). As a result, level set trees provide a means to represent and visualize data arising from complex and high-dimensional distributions that is statistically accurate in the sense of being a faithful encoding of the level sets of a *bona fide* density function. This property extends to any sub-tree of a level set tree, so that with our procedure it is possible to extract subsets of data at multiple resolutions while retaining the same probabilistic faithfulness, effectively allowing for dynamic and multi-scale clustering that does not require advance knowledge of the true number of clusters.

In the context of fiber tractography we show how the level set tree can be used interactively to visualize spatial patterns and to cluster fiber streamlines that are similar in terms of location and shape. Unlike most clustering methods that output a single partition of the data, level set trees encode many different cluster permutations and act as a scaffold for interactive exploration of clustering behavior. We also show how uncertainty can be captured on the level set tree, suggesting the potential for using the tree as a summary statistic of topographic structure. Taken together, our results demonstrate that level set trees offer a solution for describing the topographies found in fiber streamline data sets and provide a fundamentally new way of visualizing and analyzing complex spatial patterns in fiber tractography data sets.

## Methods

### Level Set Trees for Densities

Suppose we observe a collection of points 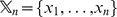 in 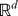 and we want to identify and visualize the spatial organization of 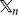 without specific knowledge about the data generating mechanism and in particular without any *a priori* information about the number of clusters. To be concrete, think of 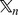 as the endpoints in 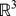 of 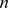 fiber pathways, which we hope to describe in a way that is anatomically meaningful. Clustering is a common approach to this goal, but clustering is typically an ill-defined task because the concept of a cluster is vaguely defined. Our level set tree methodology, in contrast, extends the statistically principled approach to clustering from Hartigan (1975) [39].

Assume the data points are independent draws from an unknown probability distribution 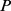 on 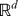 with probability density function (hereafter pdf) 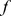. That is, 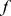 is a non-negative function such that the probability of observing a data point inside a subset 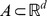 can be computed as
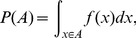
(1)where the integral is the Lebesgue integral in 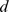-dimensions. From this expression one can see that a set 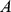 where 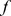 takes on large values has a high probability of containing many of the sample points. As a result, points in the sample 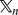 are likely clustered inside such a set, so it is natural to define clusters as regions of high density 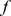.

To formalize this intuition, fix a threshold value 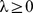 and let 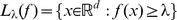 be the upper level set of 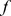, i.e. the set of points whose density values exceed the level 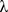. Call the set of connected components of 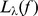 the 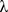-clusters of 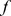. More generally, a high-density cluster of 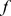 is a 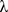-cluster for some 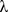, 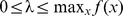. Notice that according to this probabilistic definition, the notion of a cluster depends on the choice of 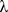 and that for a fixed 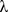 the corresponding set of clusters will typically not give a partition of 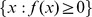. Also, for larger values of 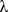 the 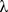-clusters define regions where the ratio of probability content to volume is higher.

A key feature of high-density clusters is the tree property: if 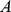 and 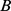 are two high-density clusters, then one is a subset of the other or they are disjoint. This implies that high-density clusters form a hierarchy–the *level set tree of* 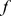
–that is indexed by the level values 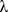. The tree property is extremely advantageous for data analysis for a number of reasons. First, the level set tree can be depicted as a dendrogram, from which the overall hierarchy of clusters of 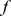 can be visualized across all possible levels of 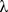. In fact, one can regard the level 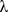 as providing a clustering resolution of sorts, with lower values of 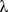 corresponding to larger and coarser clusters and higher values to smaller, more sharply defined clusters. Thus, the dendrogram of level sets of 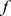 provides a multi-scale representation of the clustering characteristics of 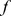. As a result, the practitioner is free to choose the scale and the number of clusters to extract, depending on the goals of the analysis. Contrast this with many popular clustering algorithms that implicitly use a single-scale approach and demand a choice of the number of clusters. Another advantage of the tree property is that it allows represention and storage of the entire set of cluster inclusions efficiently with a compact data structure that can be easily accessed and queried (see Table 1 and its description in the Results section). Finally, the dendrogram can be used in a direct and interactive manner for visualizing and extracting the clusters at various levels of the tree and for exploring the clustering features of a data set. With this approach, one can select a varying number of clusters at the same or different levels of 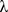 without having to re-run the algorithm.

**Table 1:** Estimated level set tree information for a simple data simulation.

Node	Start Level	End Level	Start Mass	End Mass	Size	Parent	Children
0	0.000	0.005	0.000	0.021	2001	None	[1], [2]
1	0.005	0.061	0.021	0.528	1309	0	[3], [4]
2	0.005	0.165	0.021	0.998	649	0	[]
3	0.061	0.167	0.528	0.999	359	1	[]
4	0.061	0.172	0.528	0.999	295	1	[]

**Figure 1:**
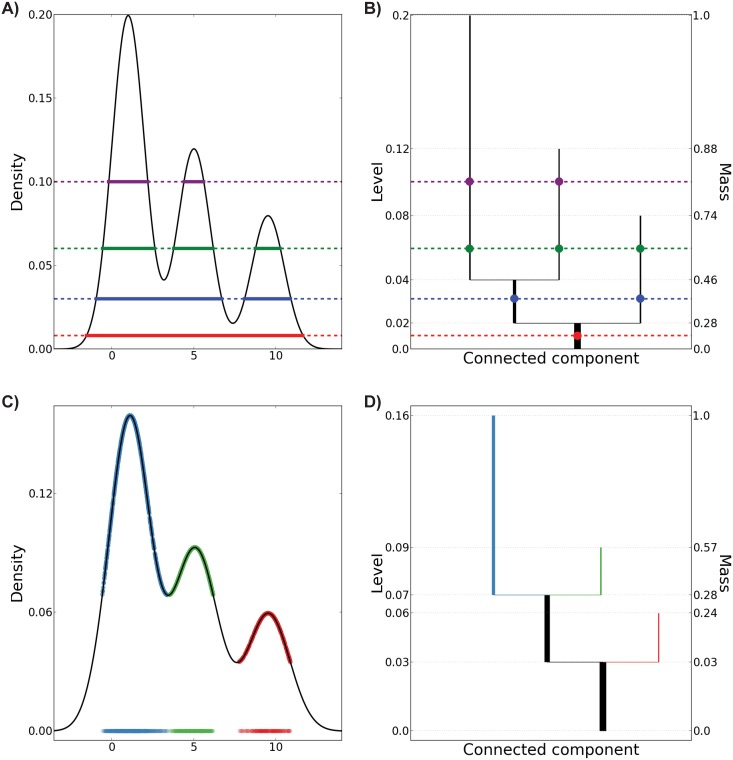
Illustration of population and sample level set trees. A) The true pdf is a mixture of three Gaussians (black curve). For each of four example density levels (dotted lines), the high-density clusters are indicated by solid line segments. B) Population level set tree for the density in panel A. The high-density clusters of panel A are found at the intersections of the selected levels (dashed lines) with the tree. C) Estimated density (black curve) based on 2,000 data points sampled from the pdf in panel A. High-density points belonging to the leaves of the sample level set tree in panel D are shown on the horizontal axis and on the estimated density function. D) Level set tree estimate based on the sample in panel C. Leaves are colored to match corresponding points in the sample. For illustration, the trees in this figure are indexed by density levels while all other trees in this article are plotted on the mass scale.

Figure 1 shows how to read and interpret the level set tree from a dendrogram. Panel A shows the pdf for a mixture of three Gaussian distributions and dashed lines representing four values of 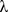. For each level the solid line segment depicts the corresponding clusters. Note that these are subsets of the real line, even though for illustrative purposes we depict them at the same level as the corresponding 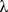. The tree property can be seen in the fact that each high-density cluster is a subset of some cluster portrayed immediately below it but is disjoint from all other clusters at the same level. In panel B the dendrogram of the level set tree is shown; note how the hierarchy of clusters corresponding to the four levels is respected. Branching points correspond exactly to levels at which two or more modes of the pdf, i.e. new clusters, emerge. Each vertical line segment in this panel represents the high-density clusters within a single pdf mode. Line segments that do not branch are considered to be high-density modes, which we call the leaves of the tree. For simplicity, we tend to treat the terms *dendrogram* and *level set tree* as synonymous.

### Estimating Level Set Trees

In practice 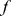 is not directly observed and one must use the data 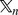 to compute an estimator 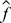 of 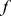. Under mild assumptions on 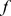 and if the sample size 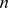 is large, 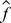 is guaranteed to be very close to 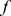 with large probability [40] and one could use the level set tree of 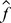 to estimate the level set tree of 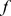 accurately. Unfortunately, computing the 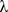-clusters of 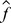 is computationally infeasible even in small dimensions because finding the connected components of the upper level sets of 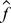 requires evaluation of the function on a dense mesh in 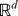 and a combinatorial search over all possible paths connecting any two points of such a mesh.

Instead, we propose a computationally tractable algorithm for level set clustering that combines and extends procedures outlined originally by Wishart (1969) [41] and more recently by Maier et al. (2009) [42], Chaudhuri and Dasgupta (2010) [43], and Kpotufe and von Luxburg (2011) [44]. At a high level, our algorithm approximates the level set tree of 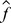 by intersecting the level sets of 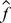 with 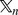 and then evaluating the connectivity of each set by graph theoretic means. The main method is outlined in Table 2: Algorithm 1, with detailed sub-procedures described in Table 3: Algorithm 3, Table 4: Algorithm 2, and Table 5: Algorithm 4. Our interactive Python toolbox for level set tree construction, analysis, and clustering is called *DEnsity-BAsed CLustering (DeBaCl)* and is available at https://github.com/CoAxLab/DeBaCl.

**Table 2:** **Algorithm 1.** Conceptual level set tree estimation procedure.

**Input**: 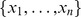
**Input: ** 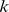
**Input** 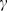
**Output:** 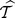 , a hierarchy of subsets of 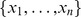 .
1: 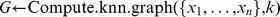
2: **for** 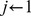 to 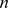 **do**
3: 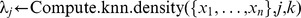
4: 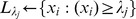 .
5: 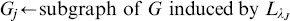
6: find the connected components of 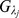 .
7: **end for**
8: 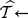 dendrogram of connected components of graphs 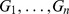 , ordered by inclusions.
9: 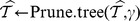
10: **return ** 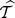

**Table 3:** **Algorithm 3.** Compute.knn.graph. Construct a k-nearest neighbor similarity graph.

**Input: ** 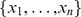
**Input: ** 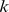
**output: ** 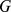 , a 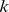 -nearest neighborhood graph.
1: **for** 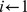 to 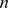 **do**
2: 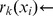 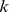 -nearest neighbor distance of 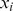 among the other sample points.
3: **end for**
4: 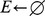
5: **for all** 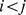 **do**
6: ** if** 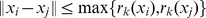 **then**
7: 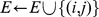
8: **end if**
9: **end for**
10: 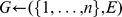
11: **return** 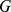

**Table 4:** **Algorithm 2.** Compute.knn.density. Compute the k-nearest neighbor density estimate at a sample point.

**Input: ** 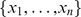
**Input: ** 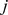 , a sample index.
**Input: ** 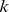
**Output: ** 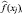 , the knn density estimate for sample point 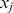 .
1: 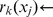 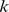 -nearest neighbor distance of 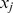 among the other sample points.
**2:** 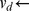 volume of the Euclidean unit ball in 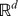 , where 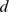 is the dimension of 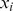 .
3: 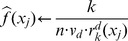
4: **return** 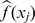

**Table 5:** **Algorithm 4.** Prune.tree. Remove small leaf nodes from the level set tree.

**Input:** 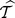 , a hierarchy of subsets of 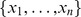 .
**Input:** 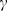
**Output:** A pruned tree 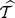
1: **for all ** 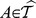 **do**
2: if 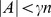 **then**
3: 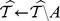
4: ** end if**
5: **end for**

The first step of our algorithm is to compute a k-nearest neighbor (knn) similarity graph 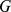 with nodes corresponding to 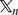 and edges connecting vertex pairs if either node is one of the 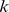 closest neighbors to the other (Table 3: Algorithm 3). In the second step, we compute a knn density estimator [42], [45], which we evaluate only at the 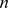 sample points (Table 4: Algorithm 2). The parameter 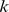 controls the smoothness of the density 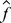; larger values of 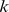 produce smoother and flatter density estimates with small variances but large biases. As a result, choosing a large 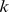 reduces the chance of finding spurious clusters but makes it harder to detect and separate true clusters that are very close to each other. Choosing a small 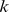 yields nearly unbiased density estimates with large variances. Based on our experiments and theoretical results ([46] and [47]), we tend to favor larger values of 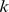.

Construction of the level set tree proceeds by ordering the estimated sample densities from smallest to largest and iterating over these values. For each value 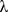 in this list, the upper level set is:
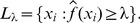
(2)In each iteration we construct an upper level similarity graph 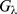 by removing the vertices from 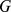 whose sample points are not in 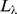, then finding the connected components of 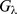.

The level set tree is the compilation of connected components over all values of 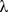. The final step of tree construction is to prune small components of the tree that occur due to sampling variability or insufficient statistical power (Table 5: Algorithm 4). Pruning merges components that contain fewer than 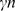 data points into other nearby components. Larger values of 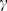 correspond to more aggressive pruning, where only connected components of large relative size are deemed as separate clusters. On the other hand, setting 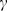 to be very small enhances the resolution of the clustering procedure but increases the chance of seeing spurious clusters.

### 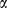-indexing

We defined the level set tree based on density thresholds 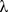. Because this indexing is highly dependent on the height of 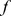 (or 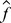), it lacks interpretability (for instance, it is not clear if 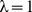 would be a threshold for high or low density regions). To remove the scale dependence, we instead consider indexing based on probability content rather than density height. Specifically, let 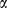 be a number between 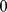 and 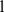 and define
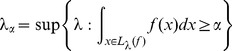
(3)to be the value of 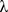 for which the upper level set of 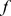 has probability content no smaller than 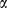 [47]. The map 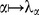 gives a monotonically decreasing one-to-one correspondence between values of 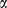 in 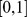 and values of 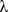 in 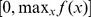. In particular, 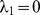 and 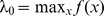. Because this map is monotonic we can define the tree in terms of the probability content 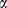 instead of 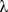 without changing the topology (i.e. number and ordering of the branches). The 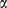-index is not a linear re-indexing of 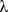, however, so the 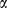-based tree is a deformation in which some branches are dilated and others are compressed. We refer to this probability-based scale as 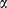
- or mass-indexing.

To estimate an 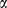-indexed tree, we index the level sets of 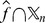 in a similar way. Specifically, for any 
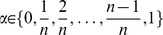
, we set 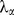 to be the 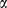-quantile of the 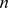 estimated sample densities. The associated hierarchy of subsets 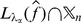 is computed as 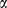 varies from 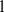 to 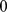.

We regard 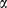-indexing as more interpretable and useful for several reasons. The 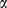 level of the tree indexes clusters corresponding to the 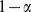 fraction of “most clusterable” data points; in particular, smaller 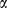 values yield more compact and well-separated clusters. The mass index can be used for de-noising and outlier removal: to eliminate 5% of the data with lowest estimated density, retrieve all the points in the clusters indexed by levels 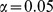. Scaling by probability content also enables comparisons of level set trees arising from data sets drawn from different pdfs, possibly in spaces of different dimensions. The 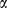 index is also more effective than the 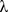 index for representing regions of large probability content but low density and is less affected by small fluctuations in density estimates.

### Pseudo-density Analysis

A fiber track can be thought of as a set of points sampled along a random curve in three dimensions. Although probability distributions for these random functions are well defined, they cannot be represented with pdfs [48]. We can extend level set trees to work with this type of non-Euclidean data by using pseudo-density functions in place of pdfs [49]. Pseudo-densities cannot be used to compute probabilities as in Equation 1, but they can be regarded as measures of similarity among points and of the overall connectivity of a space.

To compute the sample level set tree for a collection of fiber pathways, we use the knn density estimate as in Table 4: Algorithm 2 but replace the Euclidean distance with a distance relevant to fibers, expunge the term 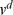 in the knn density calculation, and set 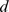 arbitrarily to 1. In general this does not yield a *bona fide* density function, but it is sufficient to induce an ordering on the data points based on each point’s proximity to its neighbors.

We measure the proximity of a pair of fibers with max-average-min distance [50], computed using the Dipy Python module’s bundles_distances_mam function [51]. Suppose a set of fiber pathways 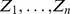, where 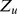 is a sequence of 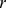 points 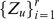, 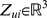. The distance between two fibers 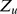 and 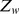 is:
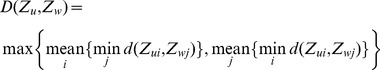
(4)where 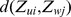 is the Euclidean distance between the 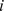
’th point in fiber 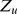 and the 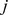
’th point of fiber 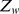. In practice, points with a small minimum distance to the other fiber are removed from the computation. Intuitively this distance matches each point in fiber 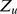 to the closest point in fiber 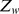 and *vice versa*, then averages the matched point distances that are sufficiently large.

Once the distance is computed for each pair of fibers, the pseudo-density function is evaluated for each fiber and a similarity graph is constructed. Level set tree construction then follows the procedure in Table 2: Algorithm 1.

### Benchmark Simulations

We compared the performance of level set trees in a traditional clustering task against several popular methods: K-means++ [52], Gaussian mixtures [53], hierarchical agglomeration with the Ward criterion [39], hierarchical agglomeration with the single linkage criterion [53], spectral [54], diffusion map [55], and DBSCAN [56]. Each method was given the true number of clusters 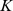 in order to isolate the effectiveness of the algorithms from the heuristics for choosing 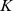. For the sake of comparison we used the *fixed* 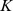 clustering option with level set trees, even though this ignores the ability of level set trees to automatically choose 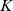.

Each method was tested in several three-dimensional data simulations with varying degrees of realism. The easiest setting was a mixture of six Gaussian distributions, the medium difficultly scenario was a mixture of three Gaussian distributions and three noisy arcs, and the most difficult test was a resampling from real fiber endpoint data. For the latter scenario, we generated data sets by resampling 5,000 points from a set of 10,000 striatal fiber pathway endpoints (from a single subject) and adding Gaussian noise. True group labels were assigned with a careful application of level set clustering. To further vary the degree of difficulty of the clustering tasks, the group means in each scenario were contracted toward the grand mean by a coefficient 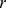, which took 20 values on a grid ranging from 0.1 to 1.2. Finally, for each simulation type and separation coefficient, we drew 20 data sets of 5,000 points each.

Both types of agglomerative hierarchical clustering were implemented with the R hclust function [57]. K-means++, Gaussian mixture modeling (GMM), and DBSCAN were implemented with the Python module scikit-learn [58]. For DBSCAN we set the neighborhood parameter to be the second percentile of all pairwise distances and the level set parameter (i.e. the number of neighbors required for a point to be a core point) to be the first percentile of pairwise distances. Note that DBSCAN does not allow 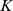 to be specified, making it difficult to compare to other methods.

We used our own implementations for spectral clustering and diffusion maps. For spectral clustering we constructed a symmetric knn graph on the data, with 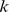 set to one percent of the sample size. The points in the first percentile of degree in this graph were removed as outliers. For diffusion maps we used a complete similarity graph with Gaussian edge weights:
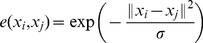
(5)with 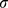 set to twice the squared median of all pairwise distances [59]. For both spectral and diffusion map clustering we used the random walk form of normalized graph Laplacian:
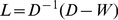
(6)where 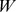 is the similarity graph adjacency matrix and 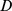 is the diagonal degree matrix for 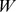 [54]. For diffusion maps the 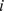
’th eigenvector 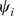 is scaled by a function of its corresponding eigenvalue 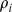:
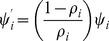
(7)which creates a multi-scale diffusion map [60]. For spectral clustering and diffusion maps we use K-means++ to cluster the data after it is projected into the eigenspace, and for spectral clustering we use a knn classifier to assign outliers to clusters.

### Comparing Whole-fiber Segmentations

To evaluate the application of the level set tree method to entire fiber streamlines (rather than streamline endpoints), we compared the clustering results for middle frontal gyrus streamlines from two subjects to the output of single linkage hierarchical clustering and K-means clustering. Both comparison methods were computed in DSI Studio (http://dsi-studio.labsolver.org). 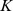 was first set to be the number of modes identified by the level set tree for each subject, then the largest 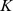 clusters were selected in both single linkage and K-means clustering. For single linkage clustering, we measured the distance between a pair of fibers 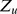 and 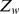 as [61]:
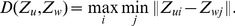
(8)The DSI Studio implementation of K-means does not use each point on fiber streamlines, but rather extracts several features: the endpoint coordinates, the middle coordinate, and the streamline length.

### Participants

Twenty male and ten female subjects were recruited from the local Pittsburgh community and the Army Research Laboratory in Aberdeen, Maryland. All subjects were neurologically healthy, with no history of either head trauma or neurological or psychiatric illness. Subjects ranged from 21 to 45 years of age at the time of scanning and four were left handed (2 male, 2 female). Participants provided written informed consent prior to participating in the study. All procedures, including the consent procedure, were approved by the Institutional Review Board (IRB) at Carnegie Mellon University.

### Imaging Acquisition

All thirty participants were scanned on a Siemens Verio 3T system in the Scientific Imaging and Brain Research (SIBR) Center at Carnegie Mellon University using a 32-channel head coil. We collected a 50 min, 257-direction diffusion spectrum imaging (DSI) scan using a twice-refocused spin-echo EPI sequence and multiple q values (TR = 9,916 ms, TE = 157 ms, voxel size  = 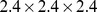 mm, FoV  = 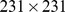 mm, b-max = 5,000 s/mm^2^, 51 slices). Minimization of head motion during acquisition was done through a custom designed setup of foam padding within the coil, designed to minimize variance of head motion along the pitch and yaw rotation directions. This setup also included a chin restraint that locked the participant’s head to the receiving coil itself. Preliminary work on resting state EPI images at the imaging center showed that this setup minimized resting head motion to about 1 mm maximum deviation for most subjects.

Scanning at multiple b-values, particularly the high b-values of DSI, can cause different distortion patterns and eddy current artifacts. Applying motion correction (which assumes that these noise sources are stationary across images) can introduce more noise into these data sets. For quality control, the diffusion weighted images were inspected before further analysis. If head motion was present in any of the diffusion weighted images (ring artifact), the whole scan section was discarded. All data used in our study passed the quality control and there was no tractable head motion in the acquired diffusion weighted images.

### Diffusion MRI Reconstruction

All DSI images were processed using a q-space diffeomorphic reconstruction method [62], implemented in DSI Studio. The co-registration was conducted using a non-linear spatial normalization approach [63], and a total of 16 iterations were used to obtain the spatial mapping function. From here orientation distribution functions (ODFs) were reconstructed to spatial resolution of 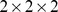 mm and a diffusion sampling length ratio of 1.25. To determine the average tractography space, we generated a template image (the CMU-30 Template) composed of the average whole-brain ODF maps across all 30 subjects. The CMU-30 Template data is available for download from the datasets page at http://www.psy.cmu.edu/~coaxlab/.

### Fiber Tractography

All fiber tracking was performed using DSI Studio. We used an ODF-streamlined region of interest (ROI) based approach [64] similar to that used in previous studies [7], [8]. Tracks were generated using an ODF-streamline version of the FACT algorithm [64]–[66]. For our initial test-set analysis, in MNI-space, we mapped two corticostriatal pathways: lateral frontal (middle frontal gyrus to striatum) and orbitofrontal (gyrus rectus to striatum). For tractography analysis on the 30 subject template brain, a whole-brain seeding was used in the tractography process, with 300 seeds per voxel in the whole-brain mask (31,100,100 total). For the fiber endpoint analysis and the test-retest analysis, we only collected 10,000 streamlines per pathway per subject. This was done to minimize processing and computational demands in the level set tree generation process and to make equivalent comparisons across pathways with the same number of samples.

Fiber progression continued with a step size of 1 mm, minimum fiber length of 10 mm, and maximum of 70 mm. To smooth each track, the next directional estimate of each voxel was weighted by 20 percent of the previous moving direction and 80 percent by the incoming direction of the fiber. The tracking was terminated when the relative quantitative anisotropy (QA) for the incoming direction dropped below a preset threshold of 0.2 or exceeded a turning angle of 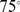. The CMU-30 template fiber pathways can be downloaded, along with a Python script illustrating level set tree estimation, at http://psy.cmu.edu/~coaxlab/data/kent_plosOne_data/.

## Results

### Visualizing Data with Level Set Trees

Table 1 displays the information in an example level set tree. The tree is a collection of nodes; each node has start and end 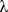 and 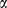 levels, a parent, children (possibly an empty set), and constituent data points at the node’s start level. This information–particularly the parent-child relationships–is conveyed more effectively with a dendrogram. Figures 1C and 1D show a density estimate for 2,000 points sampled from a mixture of three Gaussian distributions and the corresponding level set tree estimate. Each vertical line segment of the tree represents the clusters contained in a mode of the estimated pdf; all of these clusters are subsets of the cluster at the start level of the mode.

The tree visualization contains several other pieces of information. The height of each tree branch indicates the prominence of the corresponding density mode. Nodes are sorted so that density modes containing more sample points appear to the left of smaller siblings. The thickness of each tree branch and amount of surrounding whitespace are also proportional to the mass of the corresponding density mode. For example, in Figure 1D, the first split yields two nodes containing approximately 75% (black node) and 25% (red node) of the mass respectively, so the black segment is thicker and surrounded by whitespace occupying about 75% of the width of the plot.

**Figure 2:**
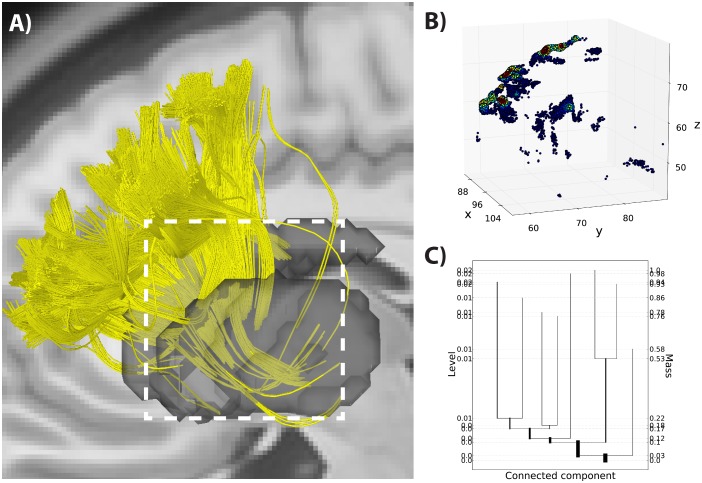
Level set tree for corticostriatal fiber endpoint locations. A) 10,000 streamlines (yellow) mapped from the lateral frontal cortex (middle frontal gyrus) to the striatal nuclei (caudate nucleus, putamen and nucleus accumbens) shown as a gray region of interest (ROI). Data taken from a representative subject. B) Endpoint locations (in millimeters) of the streamlines shown in panel A, colored by estimated density (red is high). C) The corresponding level set tree, which indicates a complex cluster structure in these data. A major split occurs when 10% of the data are excluded from the density upper level set, and each branch of the split has relevant sub-clusters at various resolutions. Note the lack of information in the density level index on this plot, which is a typical outcome.

**Figure 3:**
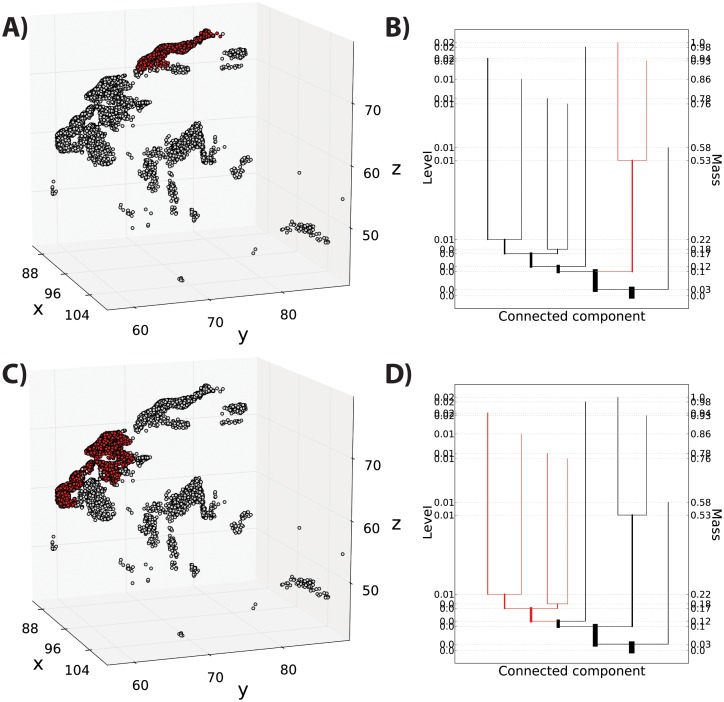
Exploring data subsets with a level set tree. A) Striatal endpoints from Figure 2. Red points are members of a selected node of the level set tree, shown in red in panel B. C) Striatal endpoints belonging to a different mode of the level set tree, shown in panel D.

The mode hierarchy shown in a level set tree is a natural platform for interactively exploring interesting subsets of complicated data; by selecting a tree branch one can zoom in on structurally coherent groups, while largely avoiding overplotting problems. Figures 2 and 3 illustrate the use of level set trees for interactive data visualization on a set of endpoint locations from 10,000 streamlines tracked from the lateral frontal cortex to the striatum. Figure 2B shows each streamline endpoint, color coded by its local density (higher densities are shown in warmer colors). The tree for this data set (Figure 2C) shows there are two primary clusters, each of which is further separated into well-defined sub-groups.

In Figure 3 we use the tree to navigate through the data. Selecting the points associated with one of the large primary branches (Figures 3A and 3B) shows that this high-density region is spatially isolated in a single cluster in the dorsal portion of the striatum, specifically the dorsal caudate nucleus. By zooming in on some of the smaller components of the other primary branch (Figures 3C and 3D) we see that these are reflected as independent sub-clusters from the first branch, with endpoints in the anterior aspect of the caudate near the shell region of the nucleus, and with local density hierarchies within the cluster (Figure 3D). This illustrates how, by interacting with the branches of the level set tree, it is possible to characterize local topographic structures at different resolutions that reflect known, anatomically distinct sub-regions of the projections into the caudate [67].

### Clustering with Level Set Trees

**Figure 4:**
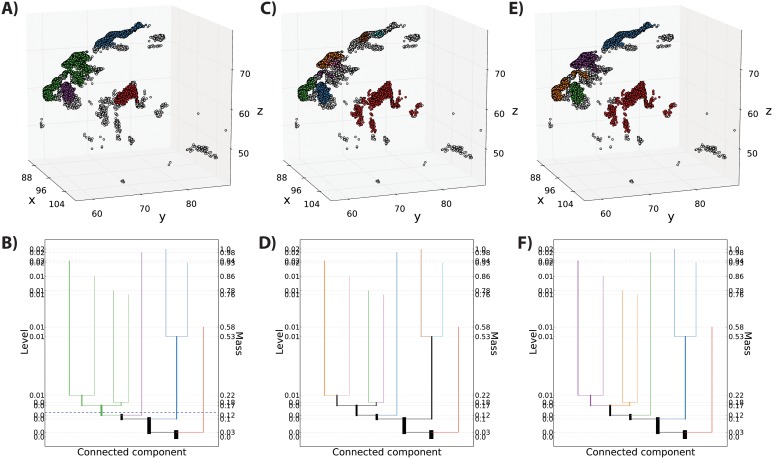
Clustering with a level set tree. A, C, E) Striatal endpoints colored by cluster assignment for three different cluster labeling methods. Gray points are unassigned because their estimated density is too low. Cluster colors match the tree node colors in the panels below. B) Tree nodes corresponding to clusters in panel A. These nodes are selected by cutting across the tree at a desired density or mass level. D) Tree nodes corresponding to clusters in panel C. Each leaf of the tree produces a cluster. F) Tree nodes corresponding to clusters in panel E. The tree is traversed upward from the root (or roots) until the desired number of clusters first appears.

Level set trees have several useful properties for solving practical clustering problems. Most notably, they provide different ways to obtain cluster labels, some of which do not require *a priori* knowledge of the number of clusters. Level set trees also identify outliers automatically and allow an investigator to visualize many different clustering permutations simultaneously and interactively. Figure 4 shows the output from three different cluster labeling methods applied to the endpoint data shown in Figures 2 and 3.

By construction, the tree is a compilation of connected components at each level of a pdf estimate, so the most straightforward cluster labeling is to retrieve the connected components at a single level (Figures 4A and 4B). In addition to its definitional nature, this method conveys the most intuitive sense for where the highest density data subsets are located. It also allows the investigator to control the number of points in the clusters; choosing a low mass level produces clusters that contain most of the data, while high mass thresholds produce clusters with only the peaks of the data modes. Finally, this method avoids the need to specify *a priori* the number of clusters, 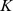, which must be determined heuristically in many popular clustering methods (K-means, for example).

The drawback of clustering at a single level is that it requires an arbitrary choice of 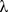 or 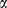. All-mode clustering, which uses each leaf node of a level set tree as a cluster, avoids this choice *and* automatically chooses the number of clusters [68] (Figures 4C and 4D). This method does remain sensitive to the choice of smoothing and pruning parameters, however. For a given degree of pruning, this method tends to produce more and smaller clusters than level set clustering.

If the clustering task demands a pre-set number of clusters, this can be done with a level set tree by identifying the first 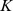 disjoint components to appear in the tree as the level increases from 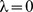. Unlike K-means (and related methods), there is no guarantee that there will be 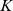 disjoint nodes in a level set tree (Figures 4E and 4F).

Each labeling method captures general streamline clusters approximately near macroscopic divisions of the striatal nuclei. For instance, the red branch in each panel of Figure 4 highlights an isolated cluster of prefrontal projections that terminate on the putamen. The remaining clusters on the caudate nucleus also break down into two major sets of endpoints. One set (dark blue in Figures 4A and 4E, brown and cyan in Figure 4C) identifies clusters of streamlines that terminate on the tail of the caudate, while the third major set (green and purple in Figure 4A; orange, green and blue in Figure 4C; orange, green and purple in Figure 4E) identifies streamlines terminating about the shell of the caudate nucleus. Thus, the first three branches of the level set tree appear to capture known anatomical sub-divisions of inputs to the striatum, with slight differences in sub-cluster identification depending on the labeling approach used.

Each of these three methods assigns cluster labels to a fraction of the sample, which we call the foreground points. The by-product of this is the intelligent removal of outliers. Figure 4 shows that the size of the foreground and outlier sets varies greatly depending on the choice of cluster labeling method and parameter values. In particular, the all-mode technique tends to create a larger number of small clusters. When a full segmentation is needed, the unlabeled background points can be assigned to a cluster with any classification technique.

Together, the advantages of a level set tree approach–avoiding the need to specify the cluster number, multiple cluster labeling methods, visualization of many cluster permutations, interactive cluster exploration, and automatic outlier identification–allow the practitioner to gain greater insight into the topography of a data set, using fewer assumptions than would be necessary for standard methods.

### Clustering Performance Evaluation

To analyze the effectiveness of level set tree clustering we tested it in a range of simulations against several standard clustering methods. The simulations ranged in difficulty over both the degree of separation of the clusters and the type of data generating process, with the most complex scenario closely mimicking fiber pathway endpoint distributions (see Methods for more detail).

**Figure 5:**
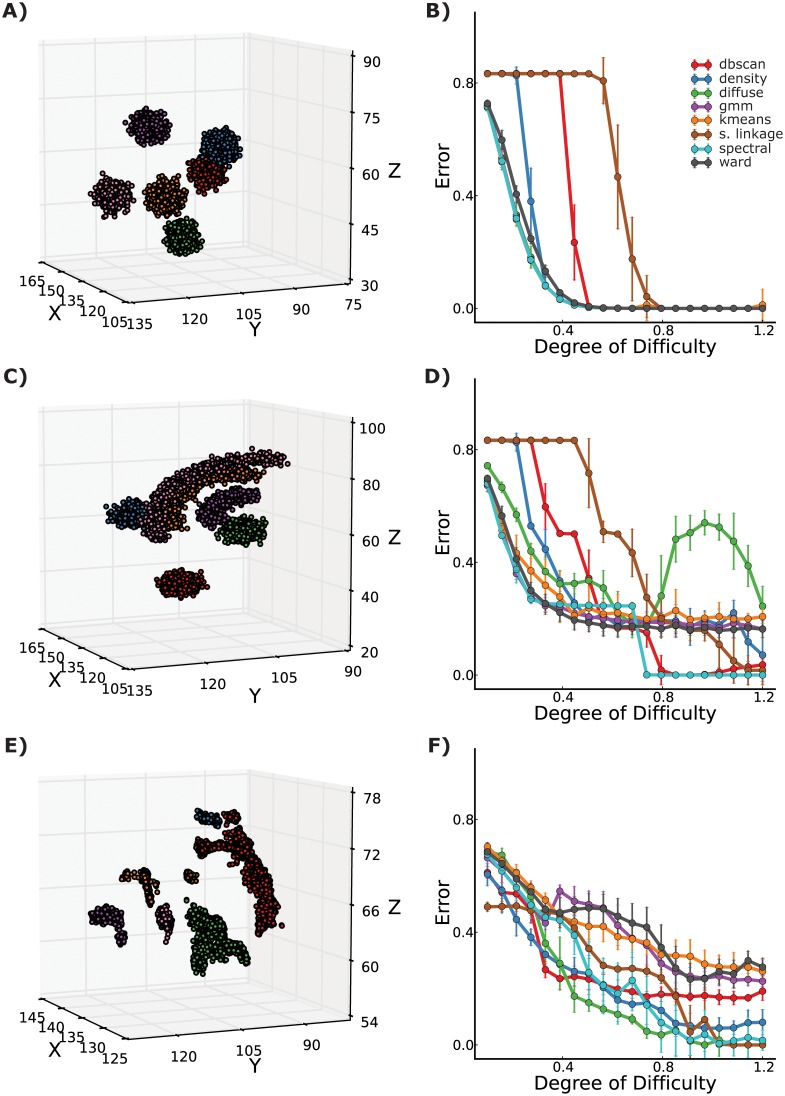
Comparison of clustering method accuracy in simulations. A, C, E) Example draws from each of three simulation scenarios (Gaussians, arcs & Gaussians, and resampled striatal endpoints, from the top), with observations colored by true group label. B, D, E) Error rate for each type of simulation over several degrees of clustering difficulty, created by contracting the groups toward the grand mean by various amounts. For each type of simulation and each degree of difficulty, the mean and standard deviation of classification error are reported for 8 clustering methods: DBSCAN (dbscan), level set tree clustering (density), diffusion maps (diffuse), Gaussian mixture models (gmm), K-means++ (kmeans), hierarchical clustering with single linkage (s.link), spectral clustering (spectral), and hierarchical clustering with linkage by the Ward criterion (ward).

Not surprisingly, for the easiest clustering task–a mixture of six spherical Gaussian distributions–all methods achieved perfect identification of the true clusters when the groups were well separated (Figure 5B). Single linkage hierarchical clustering had a very high error rate even at medium degrees of separation between clusters, due to the well-studied problem of chaining. The density-based methods DBSCAN and level set trees also required more separation between clusters before achieving the same error rate as parametric methods, possibly due to the challenge of assigning low-density points to clusters.

The results are more difficult to interpret for the moderately difficult simulation scenario with three arcs and three spherical Gaussians (Figure 5D). Single linkage hierarchical clustering again required the most separation between clusters to achieve highly accurate classification. Spectral clustering was perfect when the clusters were well separated and was as good as any other method when the clusters were very close, but performed poorly at mid-range degrees of separation. The closely related technique of diffusion maps actually became less accurate at large degrees of separation. Level set tree clustering performed poorly for tightly packed clusters, but was comparable to the parametric methods (K-means++, Ward linkage, and GMM) for somewhat- and very well-separated clusters.

In the most realistic setting with resampled real data, the parametric methods performed poorly, achieving only about 70% accuracy, even when the clusters were very well separated (Figure 5F). Each of the nonparametric methods (level set clustering, DBSCAN, diffusion maps, and spectral clustering) performed best at some degree of separation, making it difficult to identify clearly superior or inferior methods. DBSCAN and level set trees have accuracies somewhat less than 100% even for well-separated clusters, probably due to the problem of assigning low-density points to clusters. A more nuanced classifier for this step in level set tree clustering would likely improve the results for level set trees in particular.

Level set trees enjoy several categorical advantages over methods like spectral clustering and diffusion maps, namely a more intuitive representation of data structure, facilitation of interactive data exploration, a concise representation of many different clustering permutations, and automatic selection of the number of clusters. This experiment suggests level set trees are also at least as accurate in practical clustering tasks, particularly with challenging non-convex clusters.

Finally, we note that we made no attempt to choose the parameter 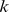 in an optimal manner in our experiments.

### Whole Fiber Segmentation

So far our analysis has focused on level set trees estimated for fiber pathway endpoints, rather than entire fiber pathways. This ignores the rich data contained in the rest of each streamline, data that can provide substantially more information about differences between sets of fibers. To work with whole fiber pathways, we adopted the pseudo-density approach, where the pairwise max-average-min fiber distance was used to rank each streamline according to the spatial proximity of its neighbors (see Methods).

**Figure 6:**
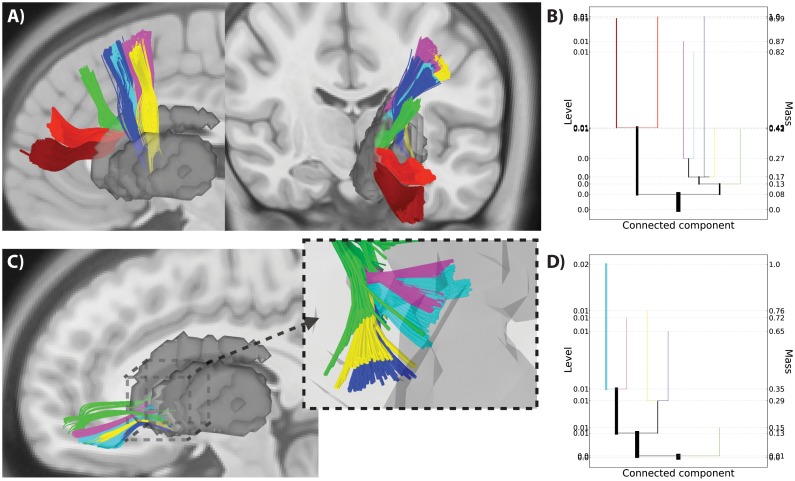
Level set tree clustering for whole fiber streamlines. **A**) Foreground fibers for the seven selected clusters from the 30 subject template data set for streamlines tracked between the middle frontal gyrus and striatum, shown in both a sagittal and coronal view. Clusters are colored according to an all-mode clustering of the tree. B) The level set tree for data in panel A. Tree leaves are matched to fiber clusters by color. C) Same analysis as shown in A, but for a set of streamlines from the orbitofrontal cortex. Inset shows closeup of fiber streamlines in the striatal ROI mask. D) Level set tree for data shown in panel C. The branch colors of trees in panels B and D match the clusters shown in the streamlines of panels A and C respectively.

Using this method, we looked at the organization of corticostriatal projections from two areas, the lateral frontal cortex and orbitofrontal cortex, in the 30 subject template brain (Figure 6). In the lateral frontal cortex we detected seven clusters of streamlines (34,982 foreground fibers out of 51,126 total fibers) that were organized in a consistent, evenly spaced rostral-caudal direction along the middle frontal gyrus (Figure 6A), an organization that is consistent with previous reports in both the animal and human literatures [8], [67], [69]. Each identified cluster reflects regions of high pseudo-density along the middle frontal gyrus. It is important to note that this whole-fiber clustering was able to capture divergent patterns in the white matter pathways. The dark blue and cyan streamlines start in the same region of the middle frontal gyrus, but diverge to different sub-cortical targets (namely, the caudate and putamen). This split is easy to identify in the level set tree by the emergence of an early branching in the tree into two major divisions that reflect caudate versus putamen fibers (Figure 6B). This provides a clean anatomical segmentation of the fibers despite the fact that these two fiber sets start in the same region of the middle frontal gyrus.

In the projections from the orbitofrontal cortex we identified five mode clusters (Figures 6C and 6D). Close inspection of the endpoints of these streamlines in the striatum reveals that each cluster forms a striated-like pattern in the caudate that is similar to patterns previously reported in corticostriatal projections [8] (Figure 6C, inset). These striated formations are thought to reflect the modularized biochemical makeup of the striatum [70], [71]. This complex arrangement is difficult to capture with clustering methods that assume convex cluster shapes, but the whole-fiber pseudo-density clustering approach successfully extracts the patterns with minimal assumptions.

**Figure 7:**
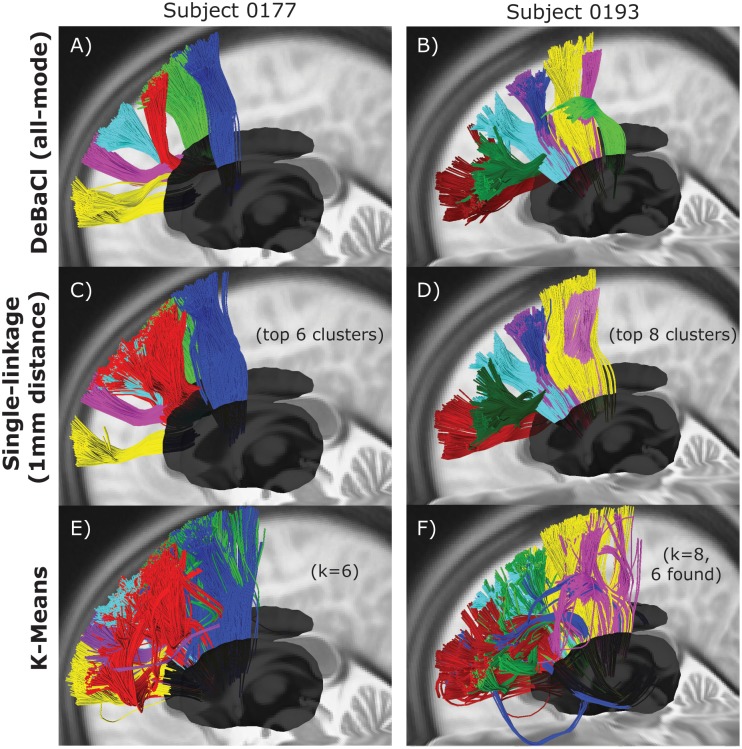
Comparison of methods for whole-fiber segmentation. A, B) High-density fiber pathway clusters from the level set tree all-mode method for middle frontal gyrus fibers in two subjects. C, D) Single linkage hierarchical clustering results for the same fiber pathways, with the dendrogram cut to match the same number of clusters in the level set tree result. E, F) K-means clustering results for the sample fiber pathways.

As with the fiber endpoint data, we also evaluated the whole-fiber level set tree results against other clustering methods: single linkage hierarchical and K-means. Figures 7A and 7B show the high-pseudo-density level set tree clusters. The single linkage method identified clusters (Figures 7C and 7D) that are similar to the high-density clusters of the level set tree result, but the boundary between clusters is less clear in both subjects (see the red, cyan and green clusters in Figure 7C and compare to Fig. 7A) for single linkage.

As expected, the K-means clustering performed much worse than the level set tree and single-linkage approaches (Figures 7E and 7F). The boundaries between the clusters are even less well defined than with single linkage, particularly for the second subject. Furthermore, there appears to be substantially larger within-cluster variation in the shape of the fiber pathways with K-means than with either of the hierarchical methods. To summarize, in qualitative terms whole-fiber level set tree clustering was able to isolate the high-pseudo-density fiber bundles as well as or better than two off-the-shelf methods.

### Level Set Tree Variability

**Figure 8:**
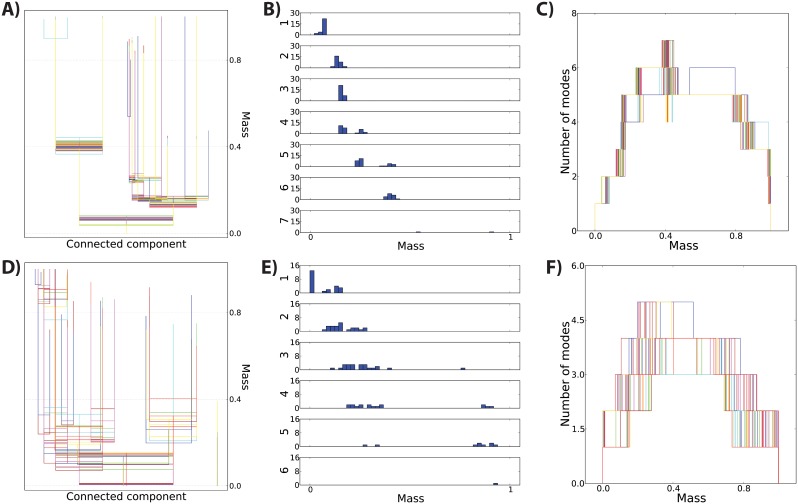
Repeat reliability for level set tree results for the 30 subject template. For the middle frontal gyrus ROI, 28 random subsamples of 15,000 fibers were drawn from the total of 51,126 fibers, while 1,500 fibers were drawn for 23 subsamples from the 3,038 total fibers in the rectus. A) All 28 level set trees plotted on the same canvas, illustrating the high degree of similarity between the data structure in the subsamples. B) Histograms of the mass levels of the splits over the whole set of subsample trees. Split mass levels are matched across subsamples by rank order. C) All 28 mode functions plotted together, illustrating that there is little variation in the number of clusters at each mass level. D) All 23 level set trees plotted together. E) Distribution of mass values for splits, matched across subsamples by rank within each sample’s tree. F) All 23 mode functions overlaid.

To assess the stability of the 30 subject template level set trees in Figure 6, we created a set of trees by subsampling from the original lateral and orbitofrontal fiber streamline data sets and constructing a tree for each subsample. This simulates the variability seen when repeating the tractography on the same data set multiple times. The overlaid tree plots in Figures 8A and 8D indicate a high degree of stability for the trees built from these subsampled data sets, although it appears there might be slightly less stability for the orbitofrontal set. This conclusion is supported for both ROIs by the mode function overlays (Figures 8B and 8E) and histograms of mass values where each tree splits (Figures 8C and 8F). These plots illustrate that the existence of each tree branch is consistent across the subsamples, although there is variation in the mass levels where the branches first appear.

The high degree of stability in these subsample trees conveys certainty to the features of the level set trees constructed on the full data set (Figures 6B and 6D). For example, the left branch of the tree for the lateral frontal projections contains two prominent nodes (red and dark red) that appear when 42 percent of the fibers are in the background (i.e. not in the the upper level set). The fact that this same split occurs in every one of the subsample trees is evidence that such a split exists in the true (but unobserved) distribution of fibers that generated this data set.

**Figure 9:**
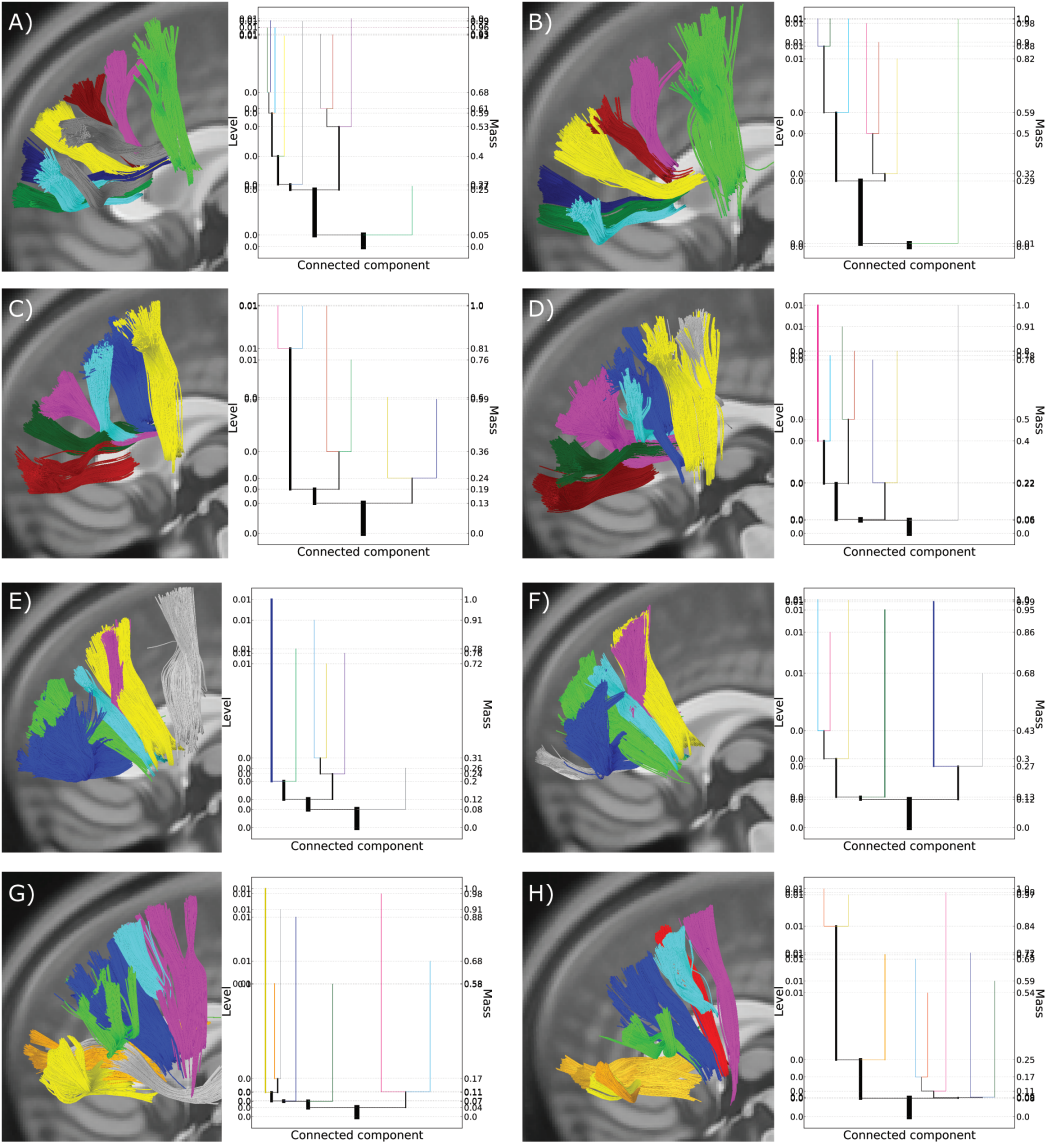
Test-retest comparisons for four subjects, tested six months apart. Colored streamlines show clusters that were consistently observed at both scan times. Gray streamlines show clusters detected at only one time point. Panels A, C, E, and G show results from the initial scan session. Panels B, D, F, and H show results from the second scanning session six months later.

On the other hand, data sets that differ even in seemingly small ways can lead to much more variation in the resulting level set trees. For a subset of subjects, fiber streamlines were reconstructed for two separate scans separated by six months. Figure 9 shows the level set trees constructed for the lateral frontal projections from each scan in several example subjects, as well as the foreground clusters produced by all-mode clustering. The foreground clusters reveal that there does tend to be an overall high degree of similarity between the fiber streamline sets across trials, with the exception of one or two well-defined clusters that only appear in one of the two scans (highlighted in gray in Figure 9).

The level set trees likewise reflect similar structure across scans, but the non-overlapping tree nodes exaggerate the apparent differences between trees. For example, panels E and F in Figure 9 show the foreground fiber streamlines and level set trees for two scans of a single subject. The blue, green, cyan, violet, and yellow clusters match well across scans and appear to share very similar topography. However, panel E contains an obvious cluster on the right side of the plot (in gray) that is not present in panel F, while panel F contains its own obvious cluster on the left side of the plot (also in gray) that is not present in panel E. Note that each branch’s (or cluster’s) color was manually defined to match between images of the same subject, but does not necessarily reflect the same branch/cluster identified across subjects. While some of the features of the trees reflect the overall similarity–for example, the number of leaves is the same and the yellow, cyan, and violet clusters are more similar to each other while the blue cluster is much different–the overall shape of the trees is very different.

These variations reflect actual differences between the test and retest data, not just variability of the level set tree procedure. Not only are some clusters present in only one of the two data sets (shown in gray in Figure 9), but differing tree shapes and branching locations indicate that the probability content and relative hierarchy of even similar-looking clusters is not the same across scans. Despite such marked differences in the test and retest data sets, the output from all-mode clustering retains a very high degree of consistency across scan sessions, demonstrating the robustness of the proposed methodology.

## Discussion

White matter pathways have highly complex shapes and spatial organization, making it difficult to summarize their topographic structure. We have shown that level set trees provide a concise representation of this topography by describing the hierarchy of modal regions in the pseudo-density function that describes the probabilistic spatial distribution of a set of fiber pathways. We demonstrated the usefulness of this hierarchy by simultaneously identifying not only major anatomical boundaries in the striatum (e.g., putamen vs. caudate), but also sub-regions within the same nucleus (e.g., shell vs. tail of the caudate; see Figure 4). Qualitative comparisons of level set trees in repeated subsampling and test-retest experiments highlight the reliability of our results and suggest that level set trees have the potential be used as statistical estimators of fiber streamline topography. Finally, we evaluated the performance of level set trees in several simulations against a suite of standard clustering methods that are commonly used to describe fiber streamline organizational patterns. Level set trees performed as well as any of the clustering methods, although we emphasize that describing fiber pathway topography is not equivalent to fiber pathway clustering. For the former purpose, level set trees have several advantages over traditional clustering techniques: they are statistically principled; they are compact data structures that enable fast retrieval of high-density clusters at any density level; they allow a multi-scale visualization of the cluster patterns in a data set; they are a natural platform for interactive data exploration; and they offer several methods for obtaining particular cluster labels without assuming the number of clusters and with automatic removal of outliers.

Level set trees are traditionally based on an estimate of an unobserved pdf that is assumed to have generated data, a realistic assumption for the endpoints of fiber pathways. We show for this type of data how level set trees can be used to visualize data patterns, interactively explore structurally coherent data subsets, and simultaneously present many different cluster labelings. Where the assumption of a pdf is not realistic, as with infinite-dimensional whole fibers, we extend the method by observing that a pseudo-density estimator (along with a similarity measure) is sufficient for level set tree construction.

Ideally, the level set tree would be used for statistical inference when comparing white matter topographies across populations. For example, one could ask if the organization of fiber streamlines between two brain areas differs in individuals with neurological disorders (e.g., autism) when compared to neurologically healthy controls. By qualitatively demonstrating the reliability of level set tree structure, we highlight this potential of the method. Quantification of the uncertainty in level set trees is an open research problem; the qualitative comparisons shown in this paper as well as other preliminary work in this direction [47], [72], [73] show that level set trees constructed on data drawn from the same distribution tend to be very similar, while trees constructed on data drawn from different distributions tend to be different. This concept of stability is difficult to apply, however, because simply identifying when two trees are similar is also an open research area (one that is beyond the scope of the current project).

An important limitation of our methodology is the selection of tuning parameters 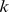 for connectivity and pseudo-density estimation and 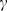 for tree pruning (although the choice of cluster number is not required as with most clustering methods). We could choose these parameters based on the optimal values found in the theoretical literature [40] but these values are only valid in asymptotic regimes and tend to work poorly in practice. As a result, as with much of applied statistics, selecting tuning parameters requires sound empirical judgment. It should be noted, however, that in our experiments the results tend to be robust for a relatively large range of tuning parameter values. Because there are several ways to obtain clusters from level set trees, inserting level set tree methods into an automated data analysis pipeline also requires a choice of cluster labeling method, in addition to the tuning parameters.

Also critical in the application of level set tree methods is the choice of function for measuring the distance between two fiber pathways. For fiber endpoints, Euclidean distance is the obvious choice, but for whole-fiber segmentation there is neither a clearly superior method nor a community-wide standard. The max-average-min distance used in this paper is popular in the fiber segmentation field [19] and performed better than several related distances in a comparison of hierarchical clustering methods in fiber tractography [18]. Intuitively, the max-average-min distance is appealing because it incorporates information from many points along each streamline without excessive influence from any single point on either pathway. The max-max-min distance (also known as Hausdorff distance), in contrast, is heavily influenced by single points that stray from the main curve of a fiber, causing fibers to cluster together only if they are extremely similar [27].

Despite these limitations, level set trees are a novel and powerful way to analyze the topography of fiber streamline data sets with minimal *a priori* assumptions. As DWI methodologies improve, the usefulness of this approach for characterizing sub-divisions in anatomical pathways will allow for greater specificity of regions of interest. Originally intended to describe probability density functions, level set trees can be extended to model pseudo-density functions as well, allowing us to apply the trees’ powerful data visualization and clustering tools in the analysis of fiber streamline data sets. This flexibility opens the door for density-based clustering approaches to be used in a variety of neuroimaging analyses beyond white matter tractography. Future work will focus on these extended applications in a neuroimaging context.
